# Albumin Suppresses Human Hepatocellular Carcinoma Proliferation and the Cell Cycle

**DOI:** 10.3390/ijms15035163

**Published:** 2014-03-24

**Authors:** Shunsuke Nojiri, Takashi Joh

**Affiliations:** Department of Gastroenterology and Metabolism, Nagoya City University Graduate School of Medical Sciences, Kawasumi 1, Mizuho-cho, Mizuho-ku, Nagoya, Aichi 467-8601, Japan; E-Mail: tjoh@med.nagoya-cu.ac.jp

**Keywords:** albumin, hepatocellular carcinoma, cell cycle

## Abstract

Many investigations have revealed that a low recurrence rate of hepatocellular carcinoma (HCC) is associated with high serum albumin levels in patients; therefore, high levels of serum albumin are a major indicator of a favorable prognosis. However, the mechanism inhibiting the proliferation of HCC has not yet been elucidated, so we investigated the effect of serum albumin on HCC cell proliferation. Hep3B was cultured in MEM with no serum or containing 5 g/dL human albumin. As control samples, Prionex was added to generate the same osmotic pressure as albumin. After 24-h incubation, the expressions of α-fetoprotein (AFP), p53, p21, and p57 were evaluated with real-time PCR using total RNA extracted from the liver. Protein expressions and the phosphorylation of Rb (retinoblastoma) were determined by Western blot analysis using total protein extracted from the liver. For flow cytometric analysis of the cell cycle, FACS analysis was performed. The percentages of cell cycle distribution were evaluated by PI staining, and all samples were analyzed employing FACScalibur (BD) with appropriate software (ModFit LT; BD). The cell proliferation assay was performed by counting cells with using a Scepter handy automated cell counter (Millipore). The mRNA levels of AFP relative to Alb(−): Alb(−), Alb(+), and Prionex, were 1, 0.7 ± 0.2 (*p* < 0.001 for Alb(−)), and 1 ± 0.3, respectively. The mRNA levels of p21 were 1, 1.58 ± 0.4 (*p* = 0.007 for Alb(−) and *p* = 0.004 for Prionex), and 0.8 ± 0.2, respectively. The mRNA levels of p57 were 1, 4.4 ± 1.4 (*p* = 0.002 for Alb(−) and Prionex), and 1.0 ± 0.1, respectively. The protein expression levels of Rb were similar in all culture media. The phosphorylation of P807/811 and P780 of Rb protein was reduced in Alb(+). More cells in the G0/G1 phase and fewer cells in S and G2/M phases were obtained in Alb(+) than in Alb(−) (G0/G1: 60.9%, 67.7%, 61.5%; G2/M: 16.5%, 13.1%, 15.6%; S: 22.6%, 19.2%, 23.0%, Alb(−), Alb(+), Prionex, respectively). The same results were obtained in HepG2. Cell proliferation was inhibited in 5 g/dL albumin medium in both HepG2 cells and Hep3B cells in 24 h culture by counting cell numbers. The presence of albumin in serum reduces the phosphorylation of Rb proteins and enhances the expression of p21 and p57, following an increase in the G0/G1 cell population, and suppresses cell proliferation. These results suggest that albumin itself suppresses the proliferation of hepatocellular carcinoma.

## 1. Introduction

Hepatocellular carcinoma is one of the most common cancers worldwide, and ranks as the fifth most common cancer and the third most common cause of cancer death [1]. Localized hepatocellular carcinoma is treated by local therapy, and other hepatocellular carcinomas are treated by transarterial chemoembolization (TACE), transcatheter arterial infusion chemotherapy (TAI), oral medication, or radiation [2]. However, local therapy for hepatocellular carcinoma has a high incidence of intrahepatic recurrence [3]. Several measures to prevent its recurrence have been investigated. It has been reported that oral BCAA may inhibit hepatocarcinogenesis [4]. We have revealed that a low recurrence rate of hepatocellular carcinoma (HCC) is associated with high serum albumin levels in patients [5]; therefore, high levels of serum albumin are a major indicator of a favorable outcome. However, the mechanism inhibiting the proliferation of HCC has not yet been elucidated, so we investigated the effect of serum albumin on HCC cell proliferation.

## 2. Results

### 2.1. Effect of Albumin Concentrations on AFP, p21, and p57 mRNA Expression and in HepG2 and Hep3B (Figures 1–4)

**Figure 1.**
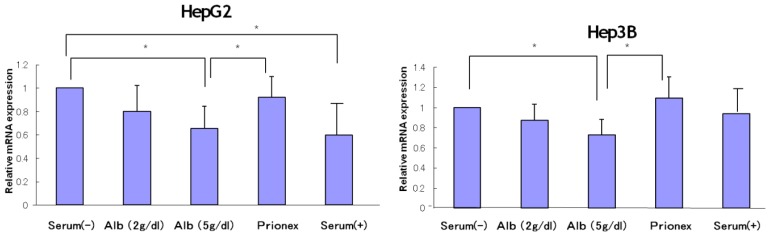
Relative mRNA expression of α-fetoprotein (AFP). mRNA levels of AFP in albumin-containing media were reduced in a concentration-dependent manner and the level in 5 g/dL albumin was significantly lower than in serum (−) and Prionex in both HepG2 and Hep3B. * *p* < 0.01.

The relative expression of AFP mRNA was significantly lower in albumin-added medium than in albumin-free medium and Prionex-added medium in both HepG2 and Hep3B cell lines in a concentration-dependent manner (Figure 1).

**Figure 2.**
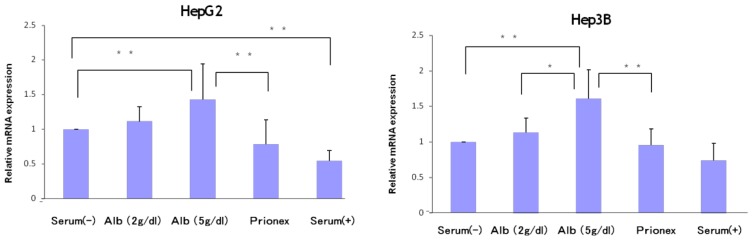
Relative mRNA expression of p21. mRNA levels of p21 in albumin-containing media were reduced in a concentration-dependent manner and the level in 5 g/dL albumin was significantly lower than in serum (−) and Prionex in both HepG2 and Hep3B. * *p* < 0.01, ** *p* < 0.001.

**Figure 3.**
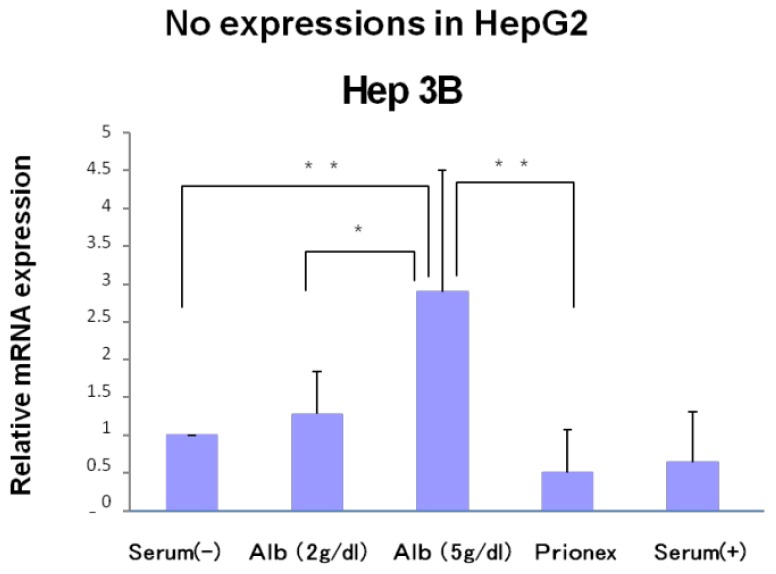
Relative mRNA expression of p57. mRNA expression in albumin-containing media was significantly higher than in serum (−) and Prionex in Hep3B in a concentration-dependent manner. mRNA expression was not seen in HepG2. * *p* < 0.05, ** *p* < 0.01.

The relative expression of p21 mRNA was significantly higher in albumin-added medium than in albumin-free medium and Prionex-added medium in HepG2 and Hep3B cell lines in a concentration-dependent manner (Figure 2), and the same result was obtained for p57 in Hep3B. p57 mRNA was not expressed in HepG2 (Figure 3). The same results were obtained even with 36 and 48 h culture so we decided to show 24 h culture results for these experiments.

**Figure 4.**
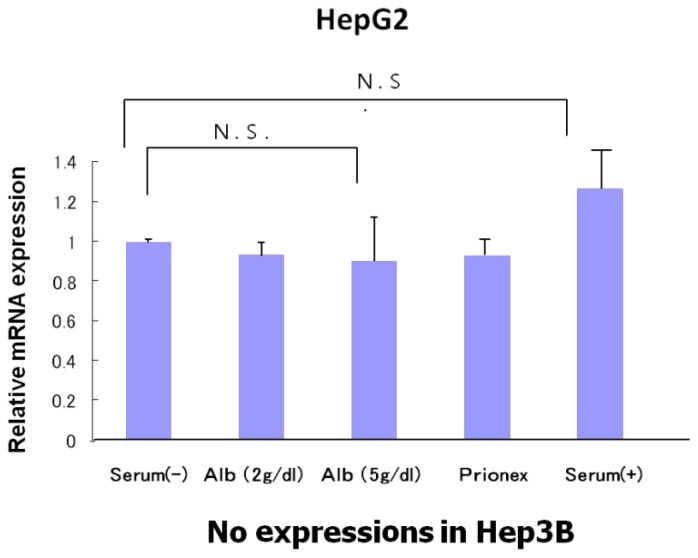
Relative mRNA expression of p53. There were no significant differences among different culture media in HepG2, and mRNA expression was not seen in Hep3B.

The relative expression of p53 showed the same level in albumin-free medium, albumin-added medium, and Prionex-added medium in HepG2 and there was no p53 expression in Hep3B (Figure 4).

### 2.2. Effect of 5 g/dL Albumin Concentrations on AFP, p21, and p57 mRNA Expressions in HepG2 and Hep3B (Figure 5)

Using media with 5 g/dL albumin, the effects of the albumin concentration on AFP, P21, P57 protein expressions were revealed. The relative expression of AFP protein expression was significantly lower in albumin-added medium than in albumin-free medium and Prionex-added medium in both HepG2 and Hep3B cell lines. We used cells cultured in serum-containing medium as a positive control and Kato III gastric cells that did not express AFP were used as a negative control. The same results were obtained for the protein level in p21 and p57 compared to mRNA expression.

### 2.3. Protein Expression of Rb and Phosphorylation of p780 and p807 (Figure 6)

Protein expression levels of Rb were similar in all culture media. However, the phosphorylation of serine 807/811 and serine 780 of Rb protein was reduced in albumin-added medium and phosphorylation was recovered in Prionex-added medium.

### 2.4. Albumin Concentration Effect on the Cell Cycle (Figure 7)

The effects of albumin concentration on the cell cycle were investigated using flow cytometry.

Cell % populations in G0/G1, G2/M, and S-phase were 45.2 ± 1.1, 43.7 ± 0.5, and 11.2 ± 1.3 in albumin-free medium, 51.2 ± 1.1, 43.1 ± 0.6, and 5.8 ± 1.8 in albumin-added medium, and 44.8 ± 0.3, 43.1 ± 0.8, and 12.1 ± 1.1 in Prionex-added medium, respectively, using HepG2.

Cell % populations in G0/G1, G2/M, and S-phase were 60.9 ± 0.6, 16.5 ± 0.9, and 22.6 ± 0.3 in albumin-free medium, 67.7 ± 1.3, 13.1 ± 0.2, and 19.2 ± 1.2 in albumin-added medium, and 61.5 ± 0.4, 15.6 ± 0.9, and 23.0 ± 0.5 in Prionex-added medium, respectively, using Hep3B.

The G0/G1 phase in albumin-added medium had a significantly higher cell population percentage than in albumin-free and Prionex-added media in both HepG2 and Hep3B cell lines (*p* <0.05).

### 2.5. Cell Proliferation in Vitro and Cell Toxicity Measurement of Albumin (Figure 8)

In 5 g/dL albumin medium, cell proliferation was inhibited more than in no-albumin media and Prionex-containing media in both HepG2 cell and Hep3B cell in 24 h culture.

We examined whether this inhibition of cell growth was due to cell toxicity derived from albumin. Cell toxicity was the same level and showed no significant changes in serum-free, albumin, and Prionex-containing media, although the toxicity tended to be lower in serum-containing media.

## 3. Discussion

Many studies have reported the inhibition of hepatocarcinogenesis [6,7]. In general, the incidence of hepatocellular carcinoma recurrence is high in patients with advanced liver fibrosis [8]. As a result, these patients have marked hypoalbuminemia. *In vitro* and *in vivo* studies have demonstrated that BCAAs promote albumin synthesis [9,10]. On the other hand, it has been reported that the mechanism of the inhibition of carcinogenesis by BCAA administration involves its immunostimulating [11,12] and antitumor [13,14] effects. The main inhibition mechanism of BCAA carcinogenesis was reported as the improvement of insulin deficiency and abnormal glucose metabolism [15,16] or its direct effects on inhibiting apoptosis induction. Although BCAAs influence pathways including mTOR, their effect can be restricted to mTOR complex 1 (mTORC1), which is associated with the translation of protein and has no effect on mTOR complex2 (mTORC2), which is closely associated with tumorigenesis [17]. The BCAA direct effect may be the induction of hepatocellular carcinoma apoptosis and the inhibition of hepatocellular carcinoma cells to inhibit the phosphorylation of GSK3β [18]. It is therefore important for the inhibition of tumor progression to inhibit only mTORC2 [19]. On the other hand, there have been no reports on the direct relationship between albumin concentrations and the inhibition of carcinogenesis. The physiological roles of albumin are to maintain colloid osmotic pressure, transport various substances, and provide an endogenous source of amino acids; however, to date, no direct inhibition of carcinogenesis by albumin has been reported. In this study, hypoalbuminemia reduced AFP expression. It is known that, although AFP is highly expressed in the fetal liver and in trace amounts in the adult liver, its expression is reactivated during hepatocyte proliferation and hepatocarcinogenesis. Since albumin inhibits AFP expression, it was expected that albumin would inhibit hepatocyte proliferation. Indeed, an experiment using flow cytometry showed that albumin significantly increased the G0/G1 phase ratio in the two cell lines, demonstrating that albumin inhibits the cell cycle. Changes in plasma osmotic pressure are known to result in changes in cell cycles and cell proliferation signals [20]. In the present experiment, the addition of the non-antigenic substance Prionex to adjust the colloid osmotic pressure eliminated the influence of changes in plasma osmotic pressure. Since albumin had a similar function in both p53-negative Hep3B cells and p53-positive HepG2 cells, these actions of albumin are not mediated by p53. Also, in HepG2 cells, albumin induced no changes in the p53 gene or protein expression levels or in the phosphorylation of various proteins (data not shown).

On the other hand, albumin inhibited the phosphorylation of Rb proteins p780 and p807/811. In addition, the expression of p21 and p57 increased significantly in the presence of albumin, suggesting that albumin induces the expression of p21 and p57 to inhibit Rb protein phosphorylation, thereby arresting the cell cycle in the G1 phase. These results suggest that albumin itself suppresses the proliferation of hepatocellular carcinoma. However, it is unclear how albumin induces the expressions of p21 and p57. It is known that, under various stress conditions, p21 induction results from p53 induction. In the present experiment, there were no changes in p53 expression or phosphorylation, and the same effect was observed in Hep3B, which did not express p53. The results revealed that these effects were associated with the Rb pathway but not with the p53 pathway.

It is generally unknown whether albumin can promote cell proliferation and this might differ among cell types. It has been reported that human or bovine albumin can promote SV-40-transformed Balb/c 3T3 cells and B cell proliferation *in vitro*, but the mechanism was unclear in these experiments [21,22] and no reports have revealed the association between albumin concentration and cell proliferation, including its biological effects. It is unclear whether albumin alone induces these changes. In the present experiment, cell cultures were washed twice with serum-free medium before the addition of albumin, and cells were cultured. However, it is unclear to what extent these growth factors remained bound to the cell-membrane surface or were secreted into the culture medium; therefore, the possibility cannot be excluded that albumin may enhance the actions of these factors.

Although further *in vivo* studies using analbuminemia rats are necessary to demonstrate the inhibition of carcinogenesis by albumin, the present study provides the first data to support the clinical observations that hypoalbuminemia is associated with a tendency to develop hepatocellular carcinoma.

## 4. Materials and Methods

### 4.1. Cell Culture

HepG2 and Hep3B hepatoma cells were grown in Dulbecco’s modified Eagle’s medium and Eagle’s minimum essential medium (MEM) containing 10% fetal bovine serum (FBS) and antibiotics. Twenty-four hours after seeding, the medium was changed to medium with no serum, containing 2 g/dL (some experiments), 5 g/dL human albumin (recombinant, Sigma, St. Louis, MO, USA) and 10% FBS for general control samples. Prionex (Polysciences, Inc., Warrington, PA, USA) was added to generate the same osmotic pressure as albumin and its data were compared. Kato III gastric cancer cells were grown in RPMI-1640 medium containing 10% FBS to use as the negative control of AFP.

### 4.2. Gel Electrophoresis and Western Blotting

After 24-h incubation with several media, cells were disrupted in lysis buffer containing 50 mM Tris (pH 8.0), 1 mM EDTA, 120 mM NaCl, 0.5% Triton X-100, 10% glycerol, 50 mmol/L NaF, 100 μmol/L Na-*O*-vanadate, 5 mmol/L Na-pyrophosphate, and protease inhibitor cocktail (Roche, Basel, Switzerland). In some experiments, the same experiments were performed using 36 and 48 h incubation and compared to 24 h. After 10 min on ice, lysates were centrifuged in the cold for 5 min and either used immediately or stored at −70 °C until use. Tyrosine phosphorylated proteins were immunoprecipitated from protein-matched lysate samples during 4-h incubation with 15 μL antiphosphotyrosine-agarose conjugate (mouse monoclonal). Immunoprecipitates were washed extensively with HNTG buffer (containing 20 mmol/L HEPES [pH 7.5], 150 mmol/L NaCl, 0.1% TritonX-100, 10% glycerol, 0.2 mmol/L sodium-*O*-vanadate, 10 mmol/L NaF, and protease inhibitor cocktail), dissolved in Laemmli buffer, and placed in a boiling water bath for 5 min. Equal volumes of each sample were separated by sodium dodecyl sulfate-polyacrylamide gel electrophoresis on 12.5% gels and transferred to PVDF membranes. After blocking with 1% BSA in TBST (10 mmol/L Tris [pH 8.0], 150 mmol/L NaCl, and 0.05% Triton X-100) for 1 h at room temperature, antiphosphotyrosine immunoprecipitates were probed with anti-p780 (BD Biosciences, San Jose, CA, USA) and p807 antibodies (mouse mixed monoclonal, 0.25 μg/mL in TBST, 60 min), followed by anti-mouse IgG-horse radish peroxidase (HRPO) (1:20,000, 30 min). Total cell lysates were directly diluted in Laemmli buffer, boiled, analyzed by gel electrophoresis, and probed with anti-AFP (Santa Cruz Biotechnology, Inc., Santa Cruz, CA, USA), P21 (BD Pharmingen, San Jose, CA, USA) antibody and anti-Rb proteins (BD Biosciences) followed by anti-mouse IgG-horseradish peroxidase, and P57 (Cell Signaling, Danvers, MA, USA) followed by anti-rabbit antibody.

After extensive washing with TBST, membranes were treated with an enhanced chemiluminescence reagent (Amersham-Pharmacia Biotech, Schenctady, NY, USA).

Bands were quantified densitometrically. Where required, the density of the phosphorylated bands and total Rb bands was normalized by comparison to β-actin (ab6276–100 antibody; Abcam, Cambridge, MA, USA) protein bands measured in the same experiment.

### 4.3. Real-Time Polymerase Chain Reaction (PCR) for Quantitative Assessment of mRNA Expression

Total RNA was extracted using Trizol reagent according to the manufacturer’s recommended protocol (Life Technologies, Grand Island, NY, USA). RNA extracts were reverse-transcribed with random hexamers and avian myeloblastosis virus reverse transcriptase using a commercial kit (Takara, Kyoto, Japan). Expressions of α-fetoprotein (AFP), p53, p21, and p57 were evaluated by real-time PCR using an ABI prism 7000 Sequence Detection system (Applied Biosystems, Tokyo, Japan) according to the manufacturer’s protocol. Probes and primers for AFP (ID: Hs00609411_m1), p53 (ID: Hs99999147_m1), p21 (ID: Hs99399142_m1), and p57 (ID: Hs00175938_m1) were purchased from Applied Biosystems. The relative target was glyceraldehyde-2-phosphate dehydrogenase (GAPDH; ID: 4352338E) mRNA in an identical cDNA sample using the standard curve method recommended by the manufacturer.

### 4.4. Cell Count and Cell Toxicity Measurement

To evaluate cell proliferation we counted cells after 24 h incubation in each solution using a Scepter handheld automated cell counter (Millipore, Billerica, Mexico). Cells were cultured in several media in a 6 cm dish and collected by cleaving with trypsin to separate cells, washing twice with PBS, resuspending cells and then counting the cells.

To estimate the cell toxicity of albumin and Prionex we measured lactate dehydrogenase (LDH) in the medium after 24-h culture using the Cyto Tox96 non-radioactive cytotoxicity assay (Promega, Fitchbarg, WI, USA), performed according to the manual.

### 4.5. Flow Cytometric Analysis

For flow cytometric analysis of the cell cycle, FACS analysis was performed as described below: after 24 h incubation, cells were washed with PBS and incubated with 0.25% trypsin for 5 min at 37 °C. After centrifuging for 5 min at 1000× *g* cells were washed with PBS, resuspended in 0.9 mL PBS, then 2.1 mL of 100% EtOH was added, and cells were vortexed. Cells were incubated for 40 min on ice. After washing cells twice with PBS, 1 mL RNse A solution was added, and cells were incubated for 30 min at 37 °C. Cells were then centrifuged for 5 min at 1000× *g*, 1 mL PI solution was added, and cells were incubated for 20 min at room temperature (RT).

The percentages of cell cycle distribution were evaluated by PI staining, and all samples were analyzed employing FACSCalibur (BD) with appropriate software (ModFit *LT*; BD, Topsham, ME, USA).

All experiments were performed using more than three individual trials and the significance of any intergroup differences was determined by ANOVA.

## 5. Conclusions

The presence of albumin in serum reduces the phosphorylation of Rb proteins and enhances the expression of p21 and p57, following an increase in the G0/G1 cell population, and suppresses cell proliferation. These results suggest that albumin itself suppresses the proliferation of hepatocellular carcinoma.

## Figures and Tables

**Figure 5.**
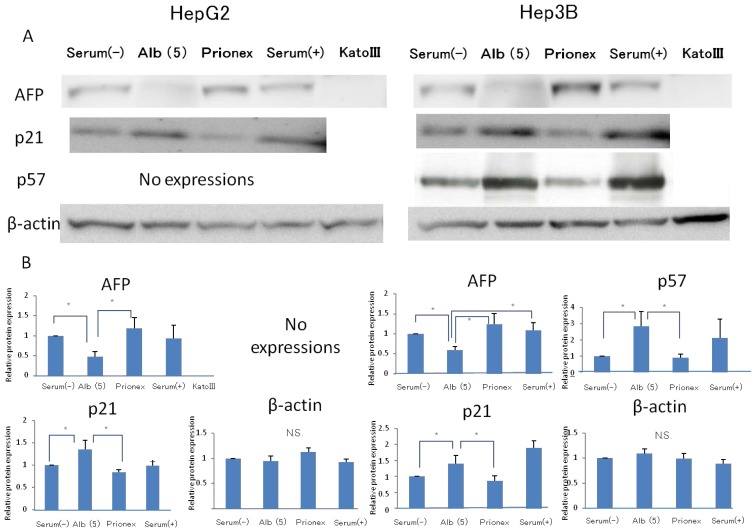
Protein expression of AFP, p21, p57. (**A**) Western blotting AFP, p21, and p57 in serum (−), 5 g/dL albumin media, Prionex, serum medium and Kato III; (**B**) Relative protein expressions were calculated in several media. Using media with 5 g/dL albumin, the effect of albumin concentration on AFP, p21, p57 protein expressions was revealed. The relative expression of AFP protein was significantly lower, and p21 and p57 were significantly higher in 5 g/dL albumin-added medium than in albumin-free medium and Prionex-added medium. Each protein was adjusted by β-actin protein. Kato III did not express AFP proteins. * *p* < 0.01.

**Figure 6.**
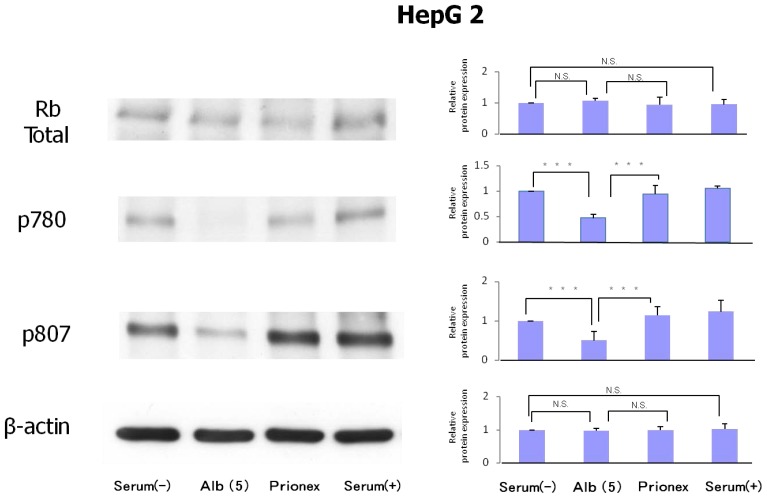
Protein expression of Rb and phosphorylation of p780 and p807. Protein expression levels of Rb were similar in all culture media. The phosphorylation of p807/811 and p780 of Rb protein was reduced significantly in 5 g/dL albumin medium. *** *p* < 0.001.

**Figure 7.**
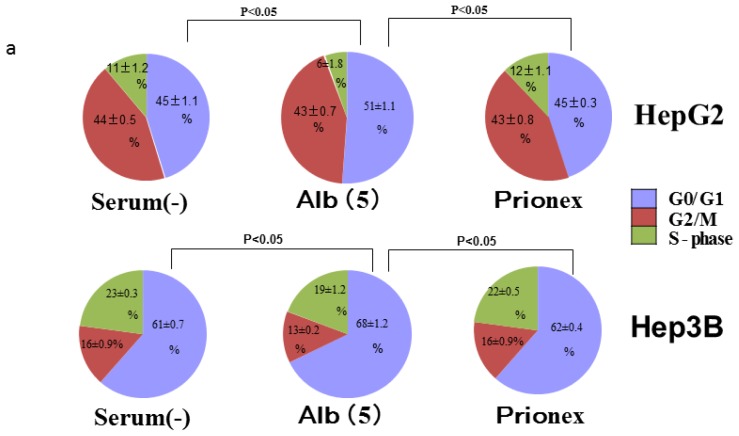

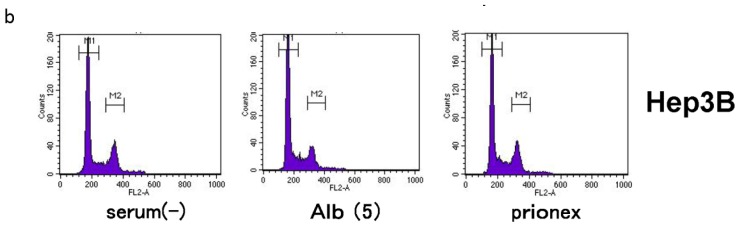
Flow cytometric analysis of the cell cycle. (**a**) More cells in the G0/G1 phase and fewer cells in S and G2/M phases were significantly obtained in Alb(+) than in serum (−) and Prionex; (**b**) Representative original histogram of cell cycle in Hep3B.

**Figure 8.**
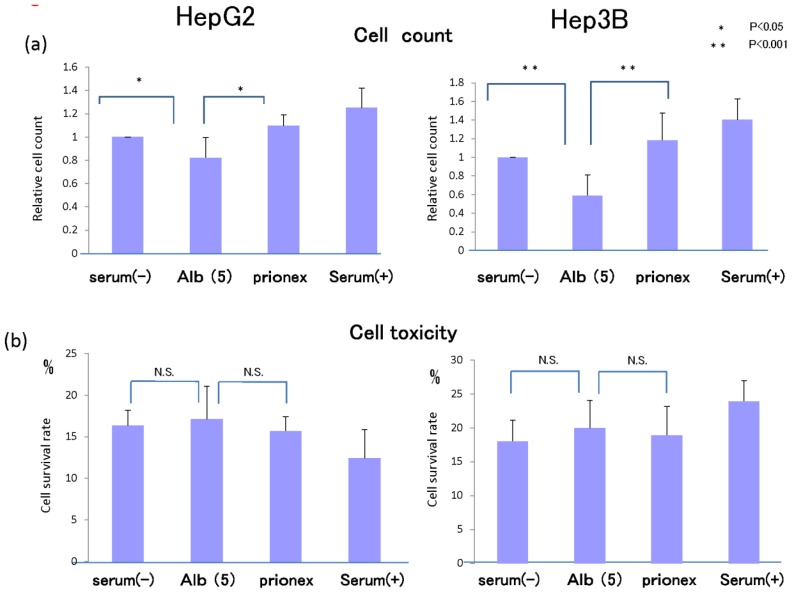
(**a**) The proliferation assay in several media. Cell proliferation was inhibited in 5 g/dL albumin medium in both HepG2 cells and Hep3B cells in 24 h culture; (**b**) Cell toxicity assay in several media. Cell toxicity was the same level and there were no significant changes in serum-free, 5 g/dL albumin, and Prionex-containing media, although the toxicity tended to be lower in serum-containing media.
